# Foreboding lncRNA markers of low-grade gliomas dependent on metabolism

**DOI:** 10.1097/MD.0000000000031302

**Published:** 2022-11-04

**Authors:** Zhuangzhuang Lu, Yugong Feng

**Affiliations:** a Department of Neurosurgery, The Affiliated Hospital of Qingdao University, Qingdao, China.

**Keywords:** lncRNA, low-grade glioma, metabolism, prognosis, signature

## Abstract

At present, there is no systematic study on the signature of long-chain noncoding RNAs (lncRNAs) involved in metabolism that can fully predict the prognosis in patients with low-grade gliomas (LGGs). Therefore, consistent metabolic-related lncRNA signatures need to be established. The Cancer Genome Atlas (TCGA) was used to identify the expression profile of lncRNAs containing 529 LGGs samples. LncRNAs and genes related to metabolism are used to establish a network in the form of coexpression to screen lncRNAs related to metabolism. LncRNA was more clearly described by univariate Cox regression. Moreover, lncRNA signatures were explored by multivariate Cox regression and lasso regression. The risk score was established according to the signature and it was an unattached prognostic marker according to Cox regression analysis. Functional enrichment of lncRNAs was shown by employing Gene Ontology (GO) and Kyoto Encyclopedia of Genes and Genomes (KEGG). Univariate Cox retrospective analysis showed that 543 metabolism-related lncRNAs were independent prognostic factors of LGG, and multivariate Cox regression analysis confirmed that 19 metabolism-related lncRNAs were prognostic genes of LGG. In the risk model, the low-risk group had a higher Overall survival (OS) than the high-risk group (*P* < .001). Univariate Cox regression analysis of risk score and clinical factors showed that risk score was an independent prognostic factor (*P* < .001, HR = 1.047, 95% CI: 1.038‐1.056). Multivariate Cox results showed that risk score could predict the prognosis of LGG (*P* < .001, HR = 1.036, 95% CI: 1.026‐1.045). ROC curve analysis showed that risk score could predict the prognosis of LGG. The areas of 1-year, 3-years, and 5 years are 0.891, 0.904 and 0.832. GO and KEGG analysis showed that metabolism-related lncRNAs was mainly concentrated in the pathways related to tumor metabolism. In order to find a more stable and reliable target for the treatment of LGG, we established 19 metabolic-related lncRNAs prognostic model, and determined that it can predict the prognosis of LGG patients. This provides a new solution approach to the poor prognosis of patients with LGG and may reverse the trend of LGG’s transformation to high-grade gliomas.

## 1. Introduction

Glioma develops from pathological glial cells, which are general tumors in the brain. Low-grade gliomas (LGGs) are a subclass of all gliomas.^[1]^ LGG is a common benign tumor, but many people are still not particularly familiar with LGGs. LGGs are a relatively mild disease, their onset rate is very slow. However, poor LGG treatment can lead to a transition to high-grade gliomas, such as glioblastoma, which can lead to a worse prognosis. The total incidence of all gliomas is 4.7 to 5.7 cases/100,000.^[2]^ The incidence of LGGs is estimated to be approximately 0.9 cases/100,000 people,^[3]^ and the typical age range of patients is from the late 30s to the mid-40s.^[4]^ The common dysfunctions of LGGs are motor impairment, cognitive impairment, and emotional disorders.^[5]^ The prognosis of LGGs is relatively good, and the survival time is usually ten years.^[6]^ The treatment plan includes observation, operation, radiotherapy, chemotherapy or comprehensive treatment, and individualized treatment is carried out according to the location, histology, molecular characteristics and patient characteristics of the tumor.^[7]^

The rapid development of cancer metabolism research areas includes sugar metabolism, lipids, amino acids and nucleic acid metabolism. To avoid a lack of metabolic resources, the loss of abnormally activated oncogenes and/or tumor suppressors keeps cancer cells in a state of constitutive intake of available glucose, glutamine and essential amino acids from the extracellular environment. In the process of tumor formation and development, metabolism undergoes obvious changes.^[8]^ A comprehensive and in-depth study of the differences in metabolites between gliomas and general tissues or different grades of gliomas has revealed their malignant behavior,^[9]^ and because metabolism plays an important role and has the possibility of therapeutic targeting, many clinical studies have identified therapeutic targets. Targeted metabolic therapy in cancer treatment has been developed for many years, and antimetabolites are still an important way to treat cancer.^[10]^

Long-chain noncoding RNAs (LncRNAs) is a noncoding RNA with a length exceeding 200 bp that is related to epigenetic regulation, alternative splicing and posttranscriptional regulation of gliomas.^[11]^ The abnormal lncRNA expression profile in clinical glioma specimens is related to the degree of malignancy and tissue differentiation, which has a high guiding significance for the subtype diagnosis and prognosis of gliomas.^[12]^ In cancer, lncRNAs can be used as both tumor suppressors and oncogenes.^[13]^ Although some lncRNAs are related to the occurrence, development and pathogenesis of gliomas, there are few studies on lncRNAs in gliomas.

The metabolic balance and energy germination of cells are maintained by glycolysis biological processes, along with oxidative phosphorylation. Dysfunctional lncRNAs regulate the above tumor metabolism characteristics. Therefore, when trying to determine effective treatment and prevention strategies in cancer progression, it is necessary to reveal the interaction between lncRNAs and cell metabolism. LncRNAs have enormous effectiveness in tumor germination, metastasis, and tumor progression. In theory, these contributions are closely related to metabolism, which can affect the adjustment of cell function.^[14]^ In previous studies, few lncRNAs, together with single lncRNAs of gliomas, were often involved. The lncRNA expression profile about metabolism in The Cancer Genome Atlas (TCGA) database has not been reported, and no new biological markers have been found to predict the prognosis of LGGs. Therefore, our goal is to use TCGA to constitute lncRNA signals and to find novel markers to forecast the outcome of LGGs.

## 2. Materials and Methods

### 2.1. Sample and dataset extraction

First, TCGA (https://portal.gdc.cancer.gov/) was used to obtain the LGG datasets for RNA sequencing (RNA-seq). TCGA database contains more than 2000 samples of 33 cancers, including transcriptome expression data, genome variation data, methylation data, clinical data and so on. It is the largest cancer gene database at present, and the genomic changes of cancer can be studied by genome analysis technology. Our study’s data criteria are listed below. Patients are diagnosed with LGG. Consummate lncRNA and clinical data must be available. Based on the inclusion criteria, 529 patients with LGG were included. Furthermore, consummate clinical data about the patient can be acquired from TCGA. When settling clinical data, less than 30 days of the follow-up period were precluded. Ethics Committee approval is not required because the information related to this research is rooted in TCGA and rigorously abides by TCGA publication regulations (http://cancergenome.nih.gov/abouttcga/policies/publicationguidelines).

### 2.2. Screening of metabolism-related lncRNAs and genes

We obtained the lncRNAs atlas from all RNAseq datasets of TCGA. The total RNA expression data were normalized by log2 transformation of scale method with “limma” R software package. Afterwards, Gene Set Enrichment Analysis (GSEA) (http://www.gsea-msigdb.org/gsea/index.jsp) was used to obtain the list of metabolism-related genes. To identify the expression of these genes in LGG, we extracted the mRNA expression matrix related to metabolism-related genes and carried out data processing. Then, the Pearson correlation method was used to count metabolism-related genes and lncRNAs to determine their relationships in LGG. LncRNAs related to metabolism were identified as the square of correlation coefficient|*R*^2^| > 0.3 and *P* < .001. Finally, we visualized the coexpression network with Cytoscape software 3.7.2 (Cytoscape Consortium, San Diego, CA).

### 2.3. Prediction of the identification of metabolism-related lncrnas

First, the metabolism-related lncRNAs’ prognostic value of overall survival (OS) was analyzed by univariate Cox regression. The *P* value is adjusted by Benjamini & Hochberg (BH). In order to reduce the risk of overfitting, we used lasso-penalized cox regression analysis to establish a prognostic model. LncRNAs were merged into selection operator (Lasso) regression, along with least absolute shrinkage, and only needed to be satisfied (*P* < .05 in univariate analysis).

Afterwards, the risk score was set up through multivariate Cox model combined with a consequence of Lasso. The independent variable in the regression is the normalized expression matrix of the candidate prognostic metabolism-related lncRNAs, and the response variable is the OS time and status of patients in the TCGA cohort. Using a linear combination of lncRNA expression level multiplied with a regression coefficient (β), we constructed a risk score: risk score $=\underset{i=1}{\overset{n}{\sum }}\;\beta_{i}*\left(expression\;of\;lncRNA_{i}\right)$. The risk score is calculated based on the normalized expression level of each lncRNAs and the corresponding regression coefficient. High-risk and low-risk groups were classified based on the median risk score. The log-rank test was used to compare survival discrepancies between the two groups.

### 2.4. Progress in the study of prognostic models

Independent prognostic models were established by Cox regression. Nomogram was used to forecast the survival time. The model’s veracity was tested by a calibration curve, along with the index of concordance (C-index). To determine whether the risk score was an unattached marker of prognosis, Cox regression analysis was integrated with demographic data. We also used “survivalROC” R package to analyze the time-varying Receiver Operating Characteristic (ROC) curve to evaluate the predictive ability of risk model. We combine risk score with age and grade to establish a nomogram to analyze the prediction ability of risk score in LGG.

### 2.5. Gene Ontology (GO) and Kyoto Encyclopedia of Genes and Genomes (KEGG) outcomes

We use “clusterProfiler” R software package to carry out GO and KEGG analysis. The expression data of gene functional enrichment are from GSEA. The top five GO and KEGG pathways interrelated to metabolism were visualized by probing the functional enrichment of lncRNAs with a predictive value.

### 2.6. Statistical analysis

Survival curves were determined by the Kaplan–Meier (KM) method, and the comparison was performed with the log-rank test. The effect of clinicopathological data, along with the lncRNA signature on prognosis, was evaluated by Lasso regression and Cox regression. R language (version 3.6), (R Core Team, New Zealand) was used to carry out statistical analysis. *P* ≤ .05 of the statistical test was statistically significant, and it was bilateral.

## 3. Results

### 3.1. Network of co-expression

In total, 14,142 lncRNAs were identified in TCGA-LGG. Thirty-four metabolism-related genes were acquired from the GSEA website, of which 33 were expressed in LGG. A lncRNA coexpression network of metabolism-related genes was constructed to identify metabolism-related lncRNAs. In the end, 4813 metabolism-related lncRNAs were selected (*P* < .001, |R^2^| > 0.3).

### 3.2. Identification of predictive lncRNA signatures

**Figure 1.**
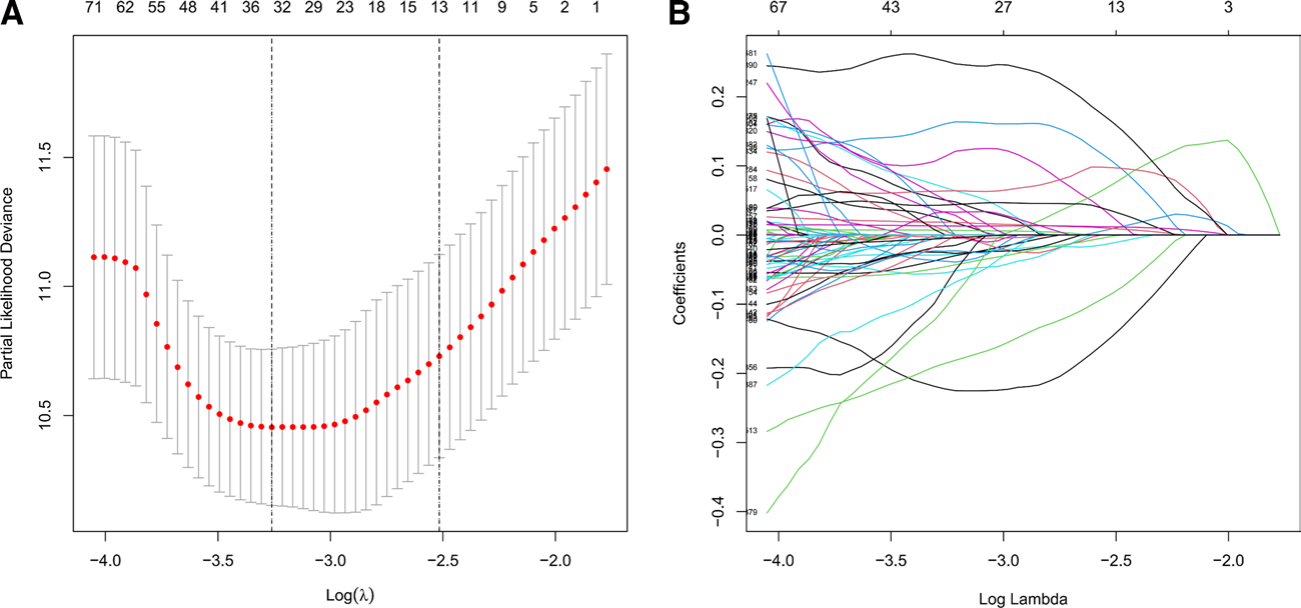
Metabolism-related lncRNAs selection utilizing the Lasso model. (a) Lasso coefficient values of 19 metabolism-related lncRNAs in low-grade gliomas (LGGs). The vertical dashed lines are at the optimal log (lambda) value. (b) Profiles of Lasso coefficients. lncRNAs = long-chain noncoding RNAs.

**Figure 2.**
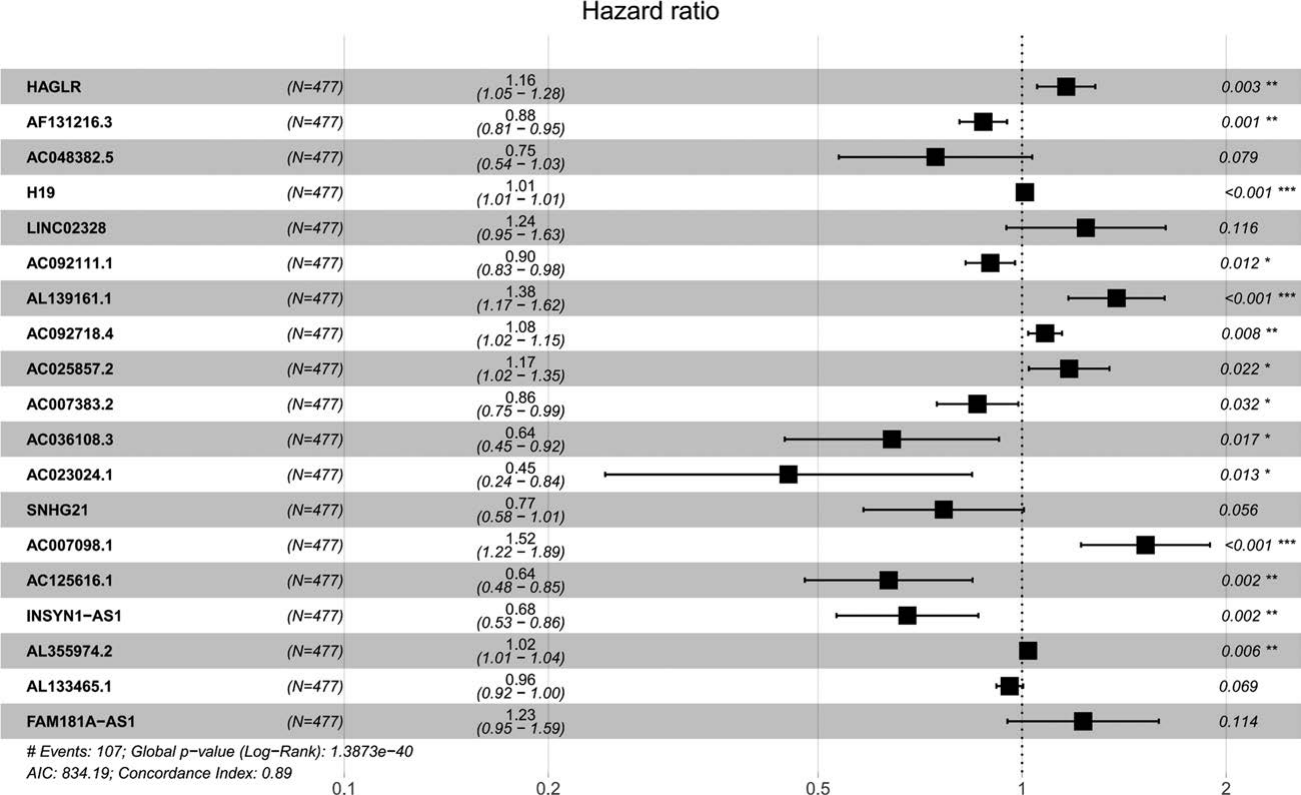
Hazard ratio (HR) value of 19 metabolism-related lncRNAs shown in the form of a forest map. lncRNAs = long-chain noncoding RNAs.

**Figure 3.**
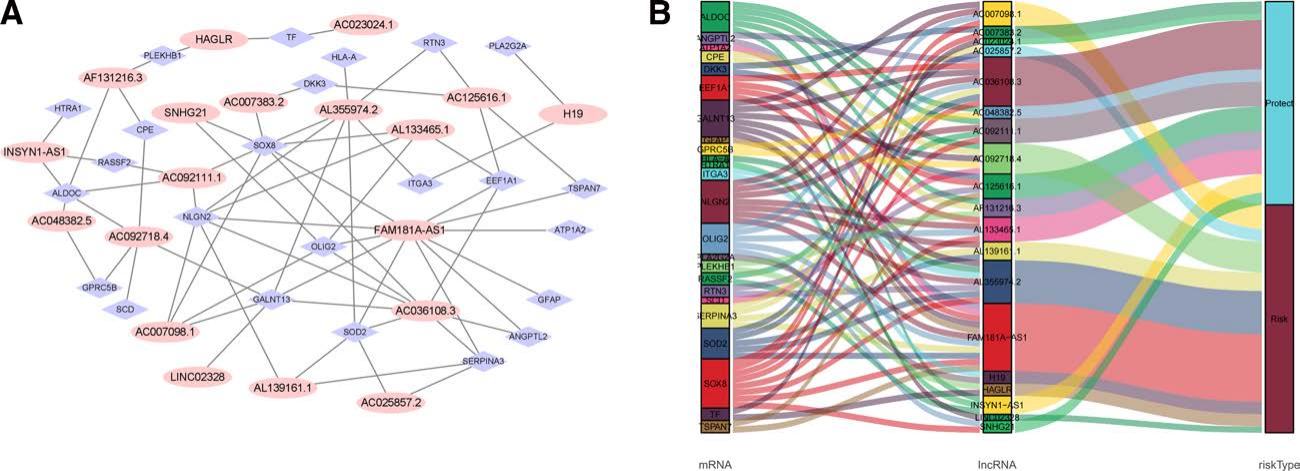
The prognostic lncRNAs’ Sankey diagram and coexpression network. (a) The coexpression network of genes and prognostic lncRNAs in LGGs. Orange ellipse nodes indicate prognostic lncRNAs, and the purple diamond nodes refer to metabolic genes. (b) Sankey diagram displaying the connection in prognostic metabolic lncRNAs, genes, and risk types. lncRNAs = long-chain noncoding RNAs, LGGs = low-grade gliomas.

**Figure 4.**
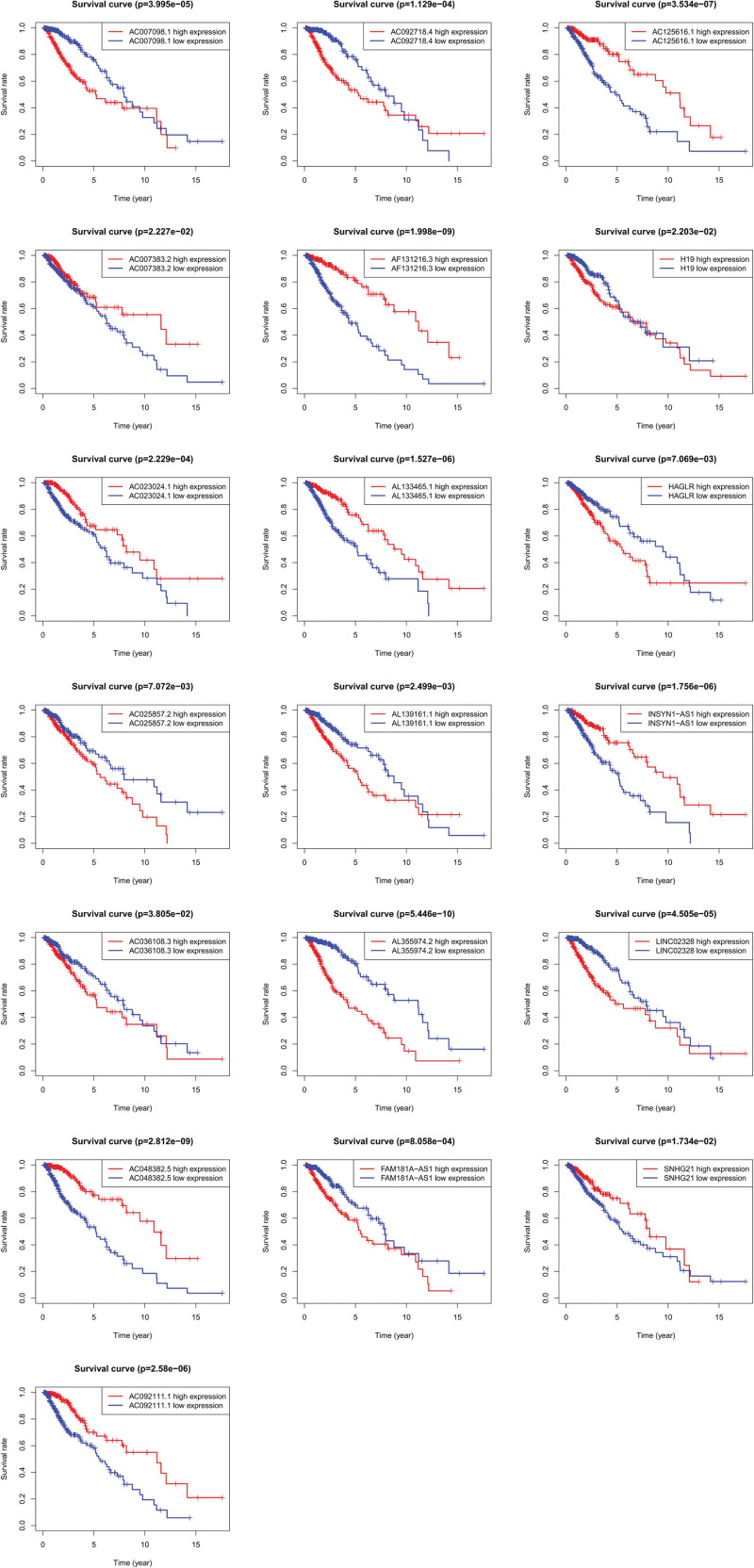
Kaplan–Meier (KM) survival curves of nineteen metabolic lncRNAs. Nine lncRNAs (HAGLR, H19, LINC02328, AL139161.1, AC092718.4, AC025857.2, AC007098.1, AL355974.2, FAM181A-AS1) were poor prognostic factors and ten lncRNAs (AF131216.3, AC048382.5, AC092111.1, AC007383.2, AC036108.3, AC023024.1, SNHG21, AC125616.1, INSYN1-AS1, and AL133465.1) were positive prognostic factors. lncRNAs = long-chain noncoding RNAs.

**Figure 5.**
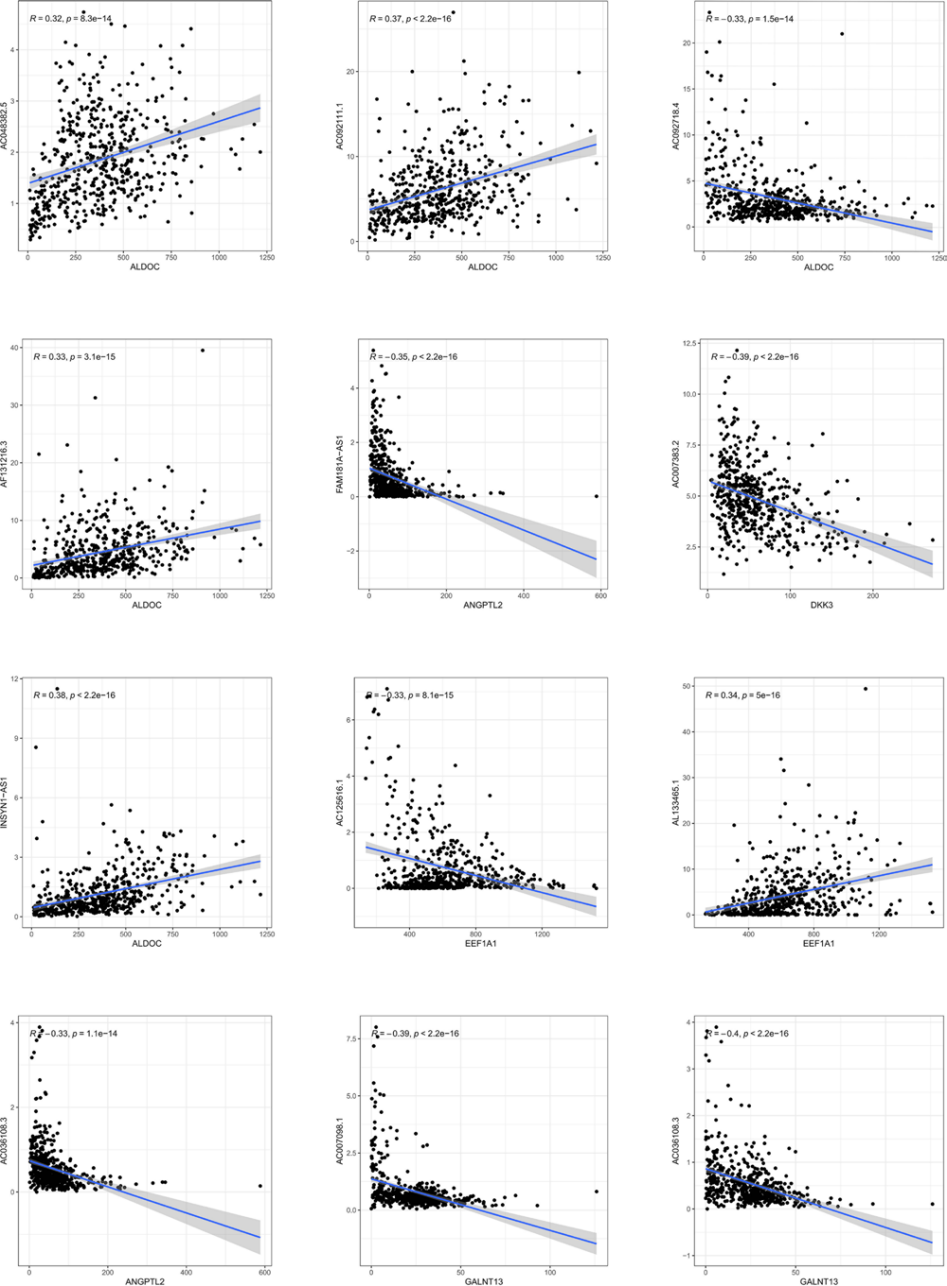
The linear relationship between metabolism-related lncRNAs and metabolism-related genes. A total of 70 results were obtained, and 12 representative results are shown here. lncRNAs = long-chain noncoding RNAs.

Cox univariate analysis showed that 543 metabolism-related lncRNAs had prognostic value in patients with LGG (*P* < .05). Afterwards, Lasso regression confirmed that there were 19 lncRNAs related to metabolism (Fig. 1), and the HR value of multivariate Cox regression analysis is shown in the form of a forest map (Fig. 2). Multivariate Cox regression analysis showed that 19 lncRNAs were independent prognostic factors (Fig. 3). Multivariate analysis showed that there were 19 prognostic factors, of which HAGLR, H19, LINC02328, AL139161.1, AC092718.4, AC025857.2, AC007098.1, AL355974.2, and FAM181A-AS1 were poor prognostic factors and AF131216.3, AC048382.5, AC092111.1, AC007383.2, AC036108.3, AC023024.1, SNHG21, AC125616.1, INSYN1-AS1, and AL133465.1 were positive prognostic factors (Table 1 and Fig. 4). These 19 lncRNAs were used to establish metabolic-related lncRNA signatures. The risk score formula is: risk score =(0.14984∗HAGLR)−(0.13184∗AF131216.3)−(0.29420∗AC048382.5)+(0.00912∗H19)+(0.21706∗LINC02328)−(0.10792∗AC092111.1)+(0.32089∗AL139161.1)+(0.07816∗AC092718.4)+(0.15983∗AC025857.2)−(0.15120∗AC007383.2)−(0.44216∗AC036108.3)−(0.79288∗AC023024.1)−(0.26622∗SNHG21)+(0.41929∗AC007098.1)−(0.45317∗AC125616.1)−(0.38951∗INSYN1−AS1)+(0.02065∗AL355974.2)−(0.04207∗AL133465.1)+(0.20741∗FAM181A−AS1). We also found a linear relationship between metabolism-related lncRNAs and metabolism-related genes. A total of 70 results were obtained, and 12 representative results are shown in Figure 5.

**Table 1:** Multivariate Cox results for lncRNAs.

lncRNA	Coefficient	HR	95% CI of HR
HAGLR	0.149	1.162	1.052‐1.282
AF131216.3	–0.131	0.876	0.808‐0.950
AC048382.5	–0.294	0.745	0.536‐1.035
H19	0.009	1.009	1.005‐1.013
LINC02328	0.217	1.242	0.948‐1.628
AC092111.1	–0.107	0.897	0.825‐0.976
AL139161.1	0.320	1.378	1.170‐1.623
AC092718.4	0.078	1.081	1.021‐1.145
AC025857.2	0.159	1.173	1.023‐1.345
AC007383.2	–0.151	0.859	0.749‐0.987
AC036108.3	–0.442	0.642	0.447‐0.924
AC023024.1	–0.792	0.452	0.243‐0.843
SNHG21	–0.266	0.766	0.583‐1.006
AC007098.1	0.419	1.520	1.222‐1.893
AC125616.1	–0.453	0.635	0.478‐0.845
INSYN1-AS1	–0.389	0.677	0.532‐0.862
AL355974.2	0.021	1.020	1.006‐1.036
AL133465.1	–0.042	0.958	0.916‐1.003
FAM181A-AS1	0.207	1.230	0.951‐1.592

CI = confidence interval, HR = hazard ratio, lncRNAs = long-chain noncoding RNAs.

### 3.3. The established signature’s influence on prognosis

**Figure 6.**
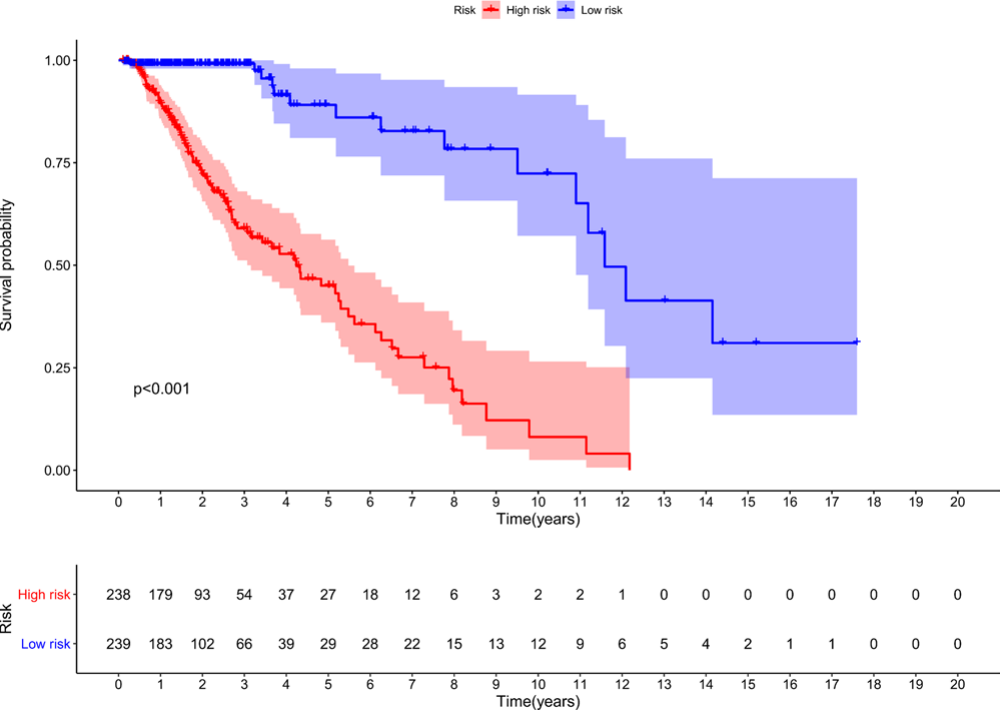
The risk score’s KM survival curve, which depended on the nineteen metabolic lncRNAs. KM = Kaplan‐Meier, lncRNAs = long-chain noncoding RNAs.

**Figure 7.**
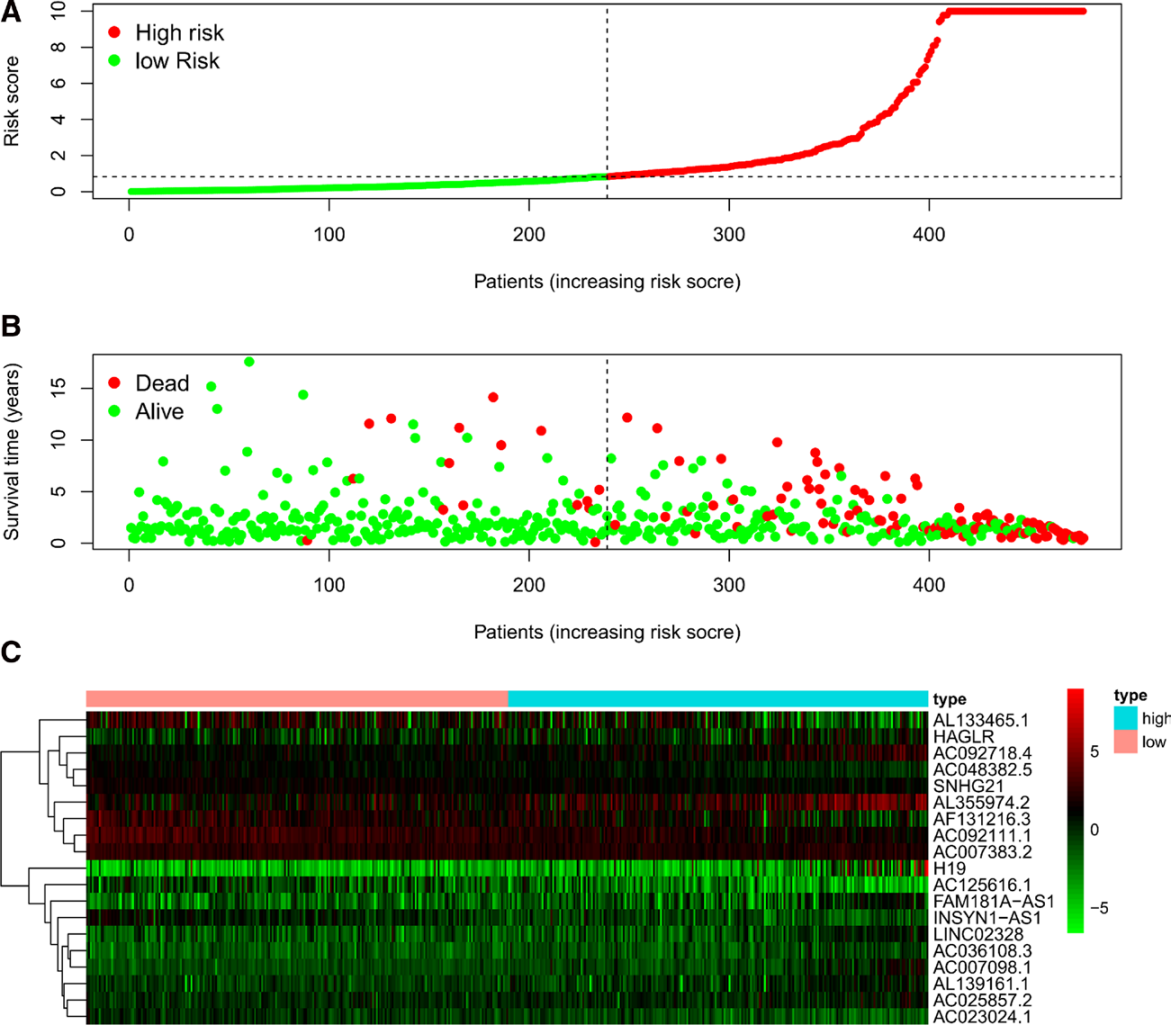
LncRNA signature for patients with LGG. (a) The risk score between the low-risk group and the high-risk group. (b) The survival time of the patients. (c) Heatmap of nineteen lncRNAs. lncRNAs = long-chain noncoding RNAs, LGGs = low-grade gliomas.

LGG patient OS was significantly correlated with the risk score of established signatures on prediction. The low-risk group had a higher OS than the high-risk group (*P* < .001) (Fig. 6). The prognosis of LGGs was significantly affected by the risk score according to Cox regression analysis (Fig. 7).

### 3.4. The lncRNA signature influence on clinical value

**Figure 8.**
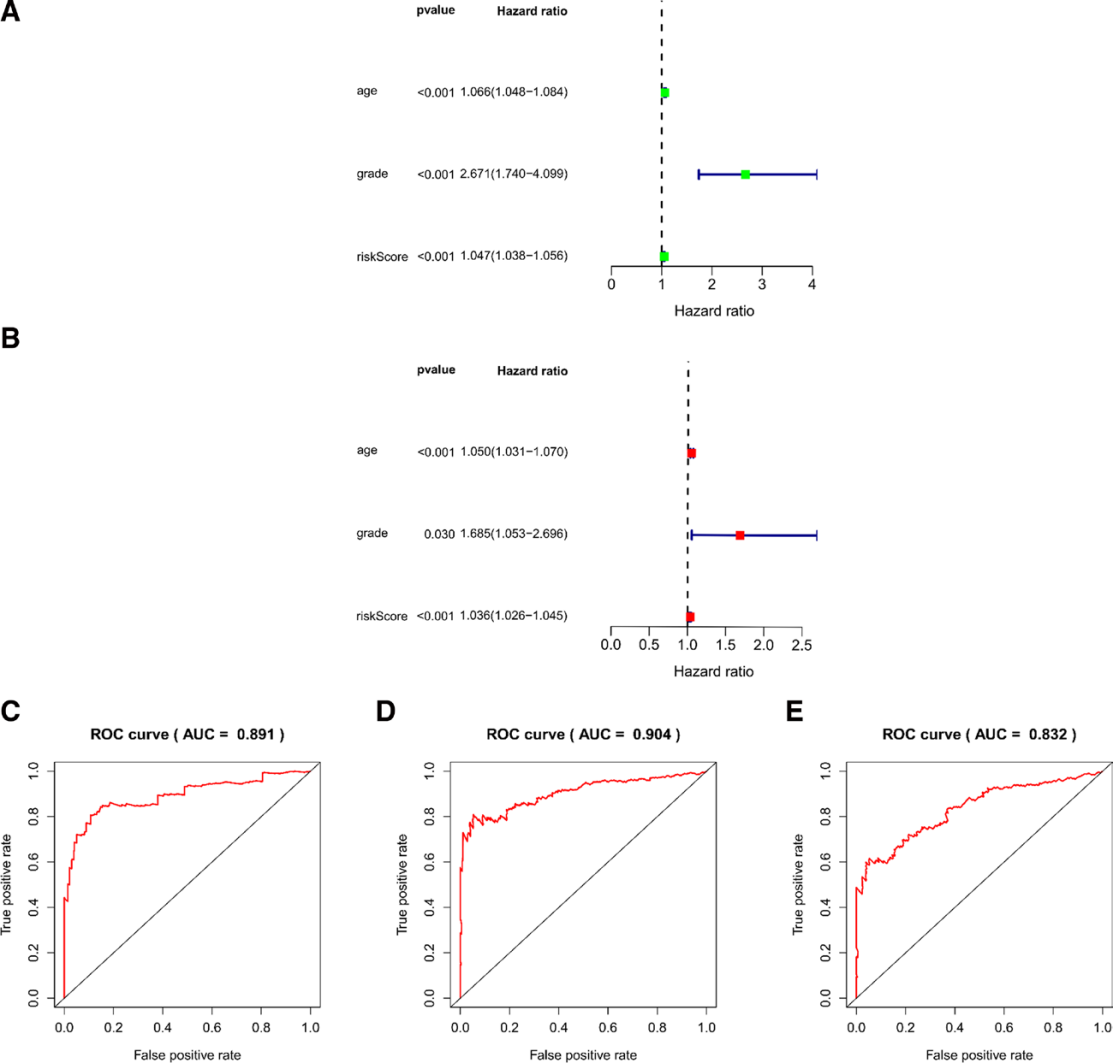
Prognostic markers based on metabolism-related lncRNAs displayed good forecasting representation. Forest plots for univariate (a) and multivariate (b) Cox regression analysis in LGG. (c‐e) The areas under the Receiver Operating Characteristic (ROC) curve at 1 year, 3 years, and 5 years. lncRNAs = long-chain noncoding RNAs, LGGs = low-grade gliomas.

**Figure 9.**
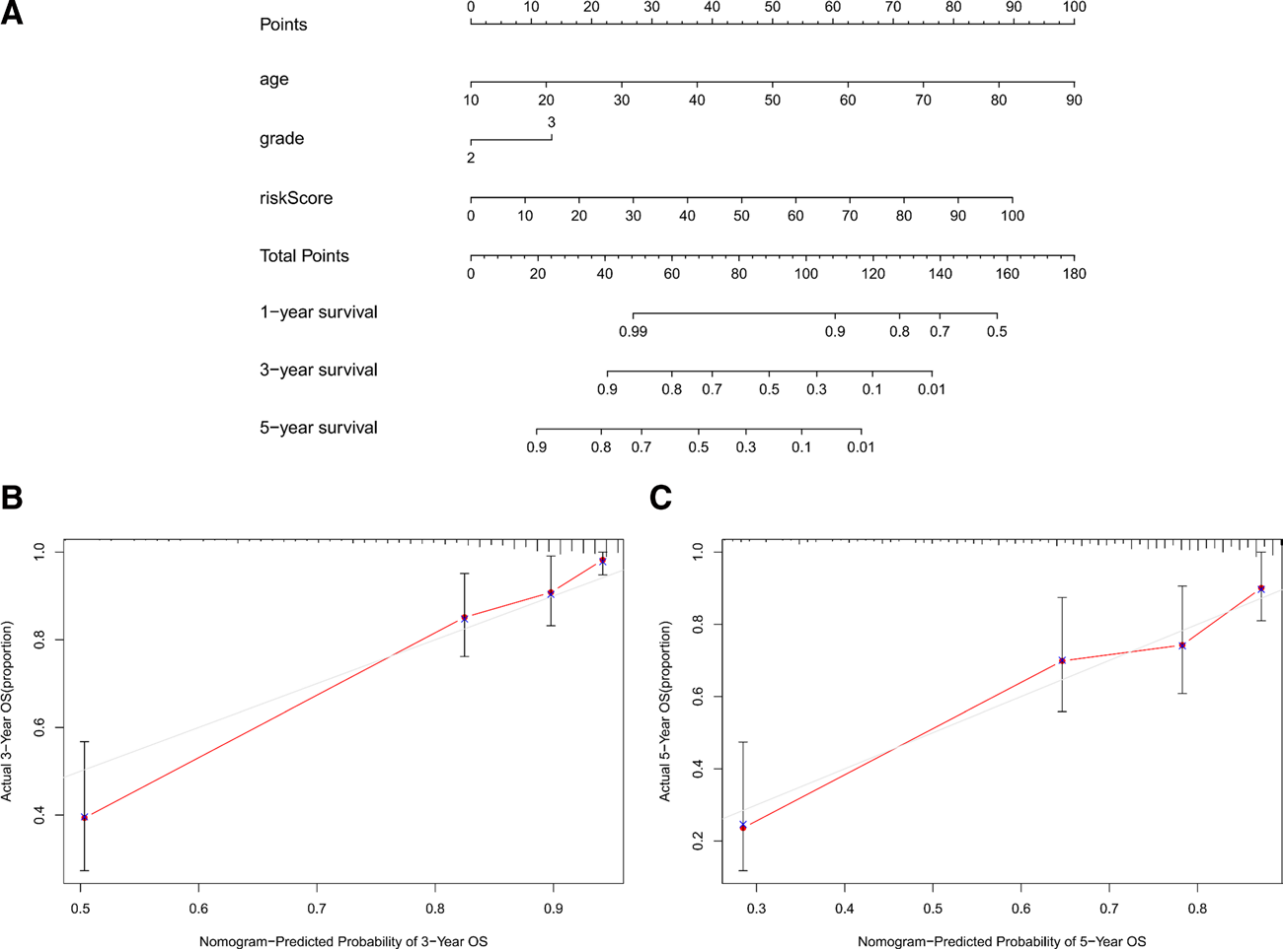
Assessment of prognostic models based on nineteen metabolism-related lncRNAs. (a) The nomogram of 1-year, 3-year or 5-year Overall Survival (OS) depended on age, grade and risk score. (b and c) The common points between the actual and predicted OS for the prognosis model were estimated by calibration curve of 3-year OS and 5-year OS. The diagonal displays reference line, where the actual probabilities are coincident with the predicted probabilities. lncRNAs = long-chain noncoding RNAs.

Three unattached prognostic markers, risk score, age and grade, were analyzed by univariate Cox regression, and the risk score’s Hazard ratio (HR) was 1.047 (*P* < .001, 95% CI: 1.038‐1.056) (Fig. 8a). Multivariate analysis also identified that the risk score was still an unattached prognostic marker after comparing the clinical features (*P* < .001, HR = 1.036, 95 % CI: 1.026‐1.045) (Fig. 8b and Table 2). The areas were 1 year (0.891), 3 years (0.904) and 5 years (0.832) under the ROC curve of survival (Fig. 8c‐e). The nomogram had age, grade and risk score, which contributions to 1-, 3- and 5-year OS, as shown in the figure (Fig. 9a). The calibration curves of 3-year OS and 5-year OS are very close to the diagonal, indicating that the prediction ability of nomogram is good (Fig. 9b and c). The prediction model showed a C-index of 0.845. The risk score increased with grade, indicating that these lncRNA signatures were interrelated with the progression of LGGs (Table 3).

**Table 2:** Clinical characteristics and risk scores of LGG using multivariate Cox regression.

Variable	B	SE	Z	HR	HR.95L	HR.95H	*P* value
Age	0.048	0.009	5.142	1.049	1.030	1.069	<.001
Gender	0.521	0.239	2.174	1.684	1.052	2.695	.030
Risk score	0.034	0.004	7.615	1.036	1.026	1.045	<.001

B = regression coefficient, HR = hazard ratio, LGGs = low-grade gliomas, SE = standard error.

**Table 3:** Clinical influences of the risk score signature for TCGA-LGG data.

Clinical	n	Risk score		*t*	*P*
Mean	SD
Age					
≤65	26	65.660	19.102	16.820	<.001
>65	437	2.505	5.276		
Grade					
2	231	1.535	4.138	-6.298	<.001
3	232	10.548	21.401		

LGGs = low-grade gliomas, TCGA = The Cancer Genome Atlas.

### 3.5. GO and KEGG outcomes

**Figure 10.**
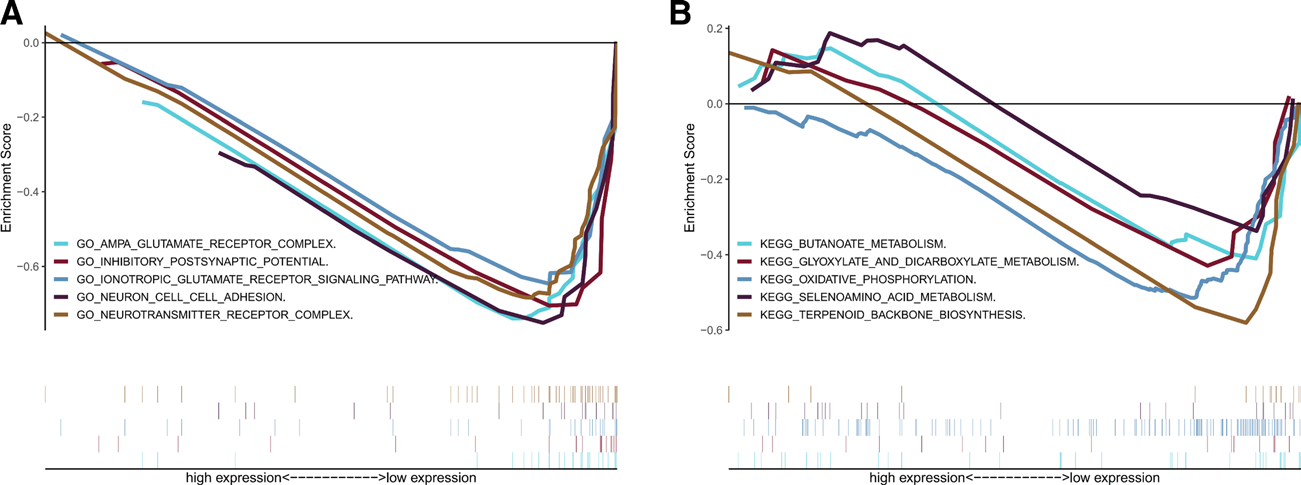
The consequence of functional analysis on account of lncRNAs. (a) Gene Ontology (GO) enrichment analysis; (b) Kyoto Encyclopedia of Genes and Genomes (KEGG) enrichment analysis. lncRNAs = long-chain noncoding RNAs.

A total of 854 GO terms and 23 KEGG pathways were acquired by functional analysis. In GO analysis, metabolic-related lncRNAs were principally concentrated in cellular components, such as the ampa glutamate receptor complex and neurotransmitter receptor complex, as well as molecular functions and biological processes, including the ionotropic glutamate receptor signaling pathway, inhibitory postsynaptic potential, and neuron cell adhesion (Fig. 10a). KEGG pathway analysis showed that lncRNAs were mainly concentrated in metabolic pathways (Fig. 10b). In addition, we found that these lncRNA sets are related to the important biological course and functional pathways of tumor formation and growth. For example, oxidative phosphorylation has an extraordinary relationship with tumor metastasis.

## 4. Discussion

Tumour can be said to be a metabolic disease. Tumour cells are able to activate specified metabolic pathways to maintain basic bioprocesses.^[15]^ Some studies have proven the importance of various lncRNAs in transcriptional interference, nucleosome modification, production and differentiation of endogenous siRNA, and transgene expression and regulation of cis, thus participating in many bioprocesses, such as cell apoptosis, proliferation and differentiation in cancer. LncRNAs exist in all kinds of human diseases and play an important role that cannot be underestimated.^[16]^ The lncRNAs have different characteristics in various tumors, reflecting the development of the disease and the prognosis of patients.^[17–19]^ The abnormal regulation of lncRNAs has also been confirmed to be interrelated with the clinical prognosis of glioma patients, suggesting that the lncRNAs can be exercised as a potential prognostic marker.^[20]^ There is solid evidence that cancer development is related to abnormal activation of signaling pathways. The role of lncRNAs in these signaling pathways has become an important part of the mechanism of carcinogenesis. Therefore, the study of lncRNAs is also expected to identify potential drugs to treat cancer. Consequently, to forecast the prognosis of patients with LGGs, it is necessary to erect a metabolically related lncRNA signature based on a large database.

A coexpression network of metabolic genes and lncRNAs was constructed to screen lncRNAs related to metabolism in our study. Furthermore, the following 19 metabolism-related lncRNAs were obtained by lasso regression and Cox regression: HAGLR, H19, LINC02328, AL139161.1, AC092718.4, AC025857.2, AC007098.1, AL355974.2, FAM181A-AS1, AF131216.3, AC048382.5, AC092111.1, AC007383.2, AC036108.3, AC023024.1, SNHG21, AC125616.1, INSYN1-AS1, and AL133465.1. These 19 metabolism-related lncRNAs could be molecular biomarkers of prognosis and larvaceous targets.

Five metabolism-related lncRNAs (HAGLR, H19, AC092718.4, FAM181A-AS1, and AC007383.2) are associated with cancer. HAGLR is upregulated in hepatocellular carcinoma and is related to proliferation and metastasis (31 cases), while the high expression of HAGLR indicates that clear cell renal cell carcinoma (ccRCC) has a good prediction.^[21]^ LncRNA HAGLR stimulates the proliferation and metastasis of liver cancer by negatively adjusting miR-6785-5p, thus aggravating the development of liver cancer.^[22]^ Lysosome-associated membrane glycoprotein (LAMP) 3 can be aggravated by HAGLR, thus enhancing epithelial-mesenchymal transition (EMT) and proliferation.^[23]^ H19 may participate in the genesis and growth of glioma and has larvaceous indicative value for glioma remission and therapy.^[24]^ LncRNA H19 has a prodigious contribution in mediating the drug counteraction of gefitinib in lung adenocarcinoma and plays a role through the H19/miR-148B-3p/DDAH1 axis.^[25]^ There is an article on AC092718.4 ‘s HR of <1, meaning that it is a positive marker of ovarian cancer (OC).^[26]^ LncRNA FAM181AAS1 is a larvaceous prognostic marker and target of glioma. The overexpression of FAM181A-AS1 significantly promoted the generation of glioma cell lines, while the glioma cell lines with FAM181A-AS1 gene knockout had the opposite effect. LncRNA FAM181A-AS1 increases the expression of ZRANB2 by sponging miR-129-5p, which promotes the growth of glioma.^[27]^ AC007383.2 has unattached prognostic value in acute myeloid leukaemia (AML) and is interrelated with tumor immunity and glutathione metabolism.^[28]^ In general, these mechanisms suggest that lncRNAs have crucial effectiveness in tumor immunity, metastasis, and metabolism, and in these processes, an imbalance in lncRNAs will make cancer cells immortal.

For the remaining 14 metabolism-related lncRNAs (LINC02328, AL139161.1, AC025857.2, AC007098.1, AL355974.2, AF131216.3, AC048382.5, AC092111.1, AC036108.3, AC023024.1, SNHG21, AC125616.1, INSYN1-AS1 and AL133465.1), their role in cancer prognosis has not been reported. Therefore, it is necessary to further study how these lncRNAs affect the prognosis of LGGs via metabolism.

The prognosis of patients with LGGs was significantly predicted based on the signature of 19 metabolic lncRNAs. The low-risk group had a higher OS than the high-risk group. The areas were 1 year (0.891), 3 years (0.904) and 5 years (0.832) under the ROC curve of survival. The risk score feature has a definite capacity in forecasting survival. Univariate Cox analysis showed that the signature could be taken as an unattached prognostic index. The model has good differentiation and veracity based on the outcomes of the ROC curve and C index, indicating that the model may be a larvaceous forecasting instrument for LGGs.

These predicted metabolic lncRNAs were obviously enriched in bioprocesses based on the results of functional enrichment analysis. In addition, in KEGG analysis, the most important pathways were enriched in the metabolic process, and we found that these lncRNA sets are related to the important bioprocess and functional pathways of tumor germination and development. There are some studies on the molecular mechanism of metabolism of LGGs. Glycolysis is a considerable marker of tumor cells and was found to be related to malignant progression in previous studies.^[29,30]^ Malignant progression and glucose metabolism in glioma cells were promoted by Pyruvate kinase isoenzyme type M2 (PKM2),^[31,32]^ while the expression of PKM2 in glioma cells was adjusted by the LINC00689/miR-338-3p axis. LncRNA has important effectiveness in LGG by regulating the transcription of Paraoxonase-2 (PON2), a gene related to energy metabolism. Overexpression of PON2 leads to a conspicuous accrescence in oxidative stress and tumor cell proliferation.^[33]^ These results prompted us to probe the mechanism of our metabolic lncRNAs.

The current research had many limitations. First, the amount of data in this study was small and only came from a database; thus, there may be errors in the analysis results. Second, this is a retrospective study that requires a prospective study to confirm the prognostic function of metabolic signals. Third, we need to further verify the stability of our model in other queues. Fourth, deeper functional experiments are required to verify the molecular mechanism of metabolism-related lncRNA effects.

## 5. Conclusion

We screened 19 metabolism-related lncRNAs related to the prognosis of LGG and established prognostic models of 19 lncRNAs composition related to metabolism. We calculated the risk score and found that the high risk group had lower OS than the low risk group. Univariate and multivariate cox regression analysis showed that risk score could be used as an independent prognostic factor. The reliability of the model is further verified by ROC curve and nomogram. Therefore, this risk model may be markers and targets for LGG. This risk model provides us with a reliable and stable solution for the treatment of LGG and fundamentally solves the problem of poor prognosis.

## Author contributions

**Conceptualization:** Zhuangzhuang Lu.

**Data curation:** Zhuangzhuang Lu.

**Formal analysis:** Zhuangzhuang Lu.

**Funding acquisition:** Zhuangzhuang Lu.

**Investigation:** Zhuangzhuang Lu.

**Methodology:** Zhuangzhuang Lu.

**Software:** Yugong Feng.

**Supervision:** Yugong Feng.

**Project administration:** Zhuangzhuang Lu.

**Resources:** Zhuangzhuang Lu.

**Validation:** Yugong Feng.

**Visualization:** Yugong Feng.

**Writing – original draft:** Yugong Feng.

**Writing – review &amp; editing:** Yugong Feng.
